# Antimicrobial and Photoantimicrobial Activities of Chitosan/CNPPV Nanocomposites

**DOI:** 10.3390/ijms232012519

**Published:** 2022-10-19

**Authors:** William M. Facchinatto, Leandro O. Araujo, Tiago B. Moraes, Thais F. Abelha, Thalita H. N. Lima, Danilo M. dos Santos, Sérgio P. Campana-Filho, Luiz A. Colnago, Anderson R. L. Caires

**Affiliations:** 1 Laboratory of Optics and Photonics, Institute of Physics, Federal University of Mato Grosso do Sul, Ave. Costa e Silva s/n, Campo Grande 79070-900, MS, Brazil; 2 Department of Biosystems Engineering, “Luiz de Queiroz” College of Agriculture, University of São Paulo, Ave. Pádua Dias 11, Piracicaba 13418-900, SP, Brazil; 3 Brazilian Corporation for Agricultural Research, Embrapa Instrumentation, St. XV de Novembro 1452, São Carlos 13560-970, SP, Brazil; 4 São Carlos Institute of Chemistry, University of São Paulo, Ave. Trabalhador São-Carlense 400, São Carlos 13560-590, SP, Brazil

**Keywords:** photoantimicrobial activity, blue-light irradiation, chitosan, CNPPV, nanocomposites, *E. coli*, *S. aureus*

## Abstract

Multidrug-resistant bacteria represent a global health and economic burden that urgently calls for new technologies to combat bacterial antimicrobial resistance. Here, we developed novel nanocomposites (NCPs) based on chitosan that display different degrees of acetylation (DAs), and conjugated polymer cyano-substituted poly(*p*-phenylene vinylene) (CNPPV) as an alternative approach to inactivate Gram-negative (*E. coli*) and Gram-positive (*S. aureus*) bacteria. Chitosan’s structure was confirmed through FT-Raman spectroscopy. Bactericidal and photobactericidal activities of NCPs were tested under dark and blue-light irradiation conditions, respectively. Hydrodynamic size and aqueous stability were determined by DLS, zeta potential (ZP) and time-domain NMR. TEM micrographs of NCPs were obtained, and their capacity of generating reactive oxygen species (ROS) under blue illumination was also characterized. Meaningful variations on ZP and relaxation time T_2_ confirmed successful physical attachment of chitosan/CNPPV. All NCPs exhibited a similar and shrunken spherical shape according to TEM. A lower DA is responsible for driving higher bactericidal performance alongside the synergistic effect from CNPPV, lower nanosized distribution profile and higher positive charged surface. ROS production was proportionally found in NCPs with and without CNPPV by decreasing the DA, leading to a remarkable photobactericidal effect under blue-light irradiation. Overall, our findings indicate that chitosan/CNPPV NCPs may constitute a valuable asset for the development of innovative strategies for inactivation and/or photoinactivation of bacteria.

## 1. Introduction

Bacteria are unicellular microorganisms present in diverse environments and involved in symbiotic relationships with a variety of other organisms [1]. However, owing to their impressive resilience and adaptability, strains of several pathogenic bacteria are continuously getting resistant to antibiotics. Recent data reveals that antibiotic-resistant bacteria are responsible for about 700,000 deaths every year, which might reach over 10 million annually, associated with a cost of 100 trillion USD, by 2050 if innovative actions are not carried out [2]. Due to a growing concern on antimicrobial resistance (AMR), the World Health Organization (WHO) has indicated an urgent need of taking immediate actions to develop novel medicines and treatments as part of its global action plan on AMR [3]. In this context, the development of new antibiotics usually figures as an unsustainable approach, as they are often related to high prices, long-term toxicity [4] and may further boost the natural selection of resistant strains [5]. Alternatively, bacterial structure disruption triggered by a photochemical mechanism [6,7] seems a feasible strategy to stop antibiotic-resistant strains from spreading.

Among the existing phototherapies used to inactivate microorganisms, antimicrobial photodynamic therapy (aPDT) has gained prominence due to its capability of inactivating multidrug-resistant bacteria [8,9,10,11]. This approach is based on the generation of free radicals or reactive oxygen species (ROS) through light-activated molecules, known as photosensitizer chemical agents (PS) [6]. In this way, bacterial photoinactivation by aPDT is mainly reached by oxidatively damaging the surface of biomolecules, blocking the process of protein synthesis or even inducing DNA mutation [12,13,14]. Its multitarget and multiaction nature is advantageous once the involved mechanism can lead to bacterial inactivation regardless of their antibiotic resistance levels [15]. However, despite this great potential, aPDT may be not efficient depending on the physicochemical features of PS water solubility, photostability, electrical charge, etc. [16]. Aiming to overcome these possible limitations, new photoresponsive agents have been proposed with special attention to nanobased PS [17,18,19]. For instance, conjugated polymer nanoparticles (CPNs) have been reported as promising candidates for aPDT [20,21,22].

Conjugated polymers (CP) have been attracting increasing interest for manufacturing organic-based photo devices, which has greatly expanded their applications for biosensing [23], bioimaging [24], antimicrobial photoinactivation [20,21,25,26,27] and theranostics [28] due to their inherent optoelectronic properties, such as charge transport and fluorescence quantum yield [29,30]. For instance, the generation of ROS by CPNs under light illumination can enhance the cytotoxicity against bacteria [31]. Furthermore, a structure modification of CPNs by functionalizing them with cationic groups may significantly improve the electrostatic interaction and disruption of bacterial surfaces with and without light irradiation [20,27]. Studies have shown that positively charged imidazolium-substituted poly(3-hexylthiophene) (P3HT-Im) grants almost 99% reduction of *S. aureus* (Gram-positive), while an 18% reduction is achieved for *E. coli* (Gram-negative) under dark conditions [32]. In contrast, cationic poly(*p*-phenylene vinylene) (PPV) functionalized with quaternary ammonium groups reduces about 70% of *E. coli* under visible light irradiation [33]. Similarly, other PPV cationic derivatives have shown binding affinity with *E. coli* surfaces, improving the PPV’s bactericidal activity [21,34].

The antimicrobial activity of cationic materials can be achieved by using chitosan, a biocompatible and biodegradable biopolymer constituted by 2-acetamido-2-deoxy-D-glycopyranose (GlcNAc) and 2-amino-2-deoxy-D-glycopyranose (GlcN) residues linked by (β → 4) glycosidic bonds, mainly due to its intrinsic polycationic character in acidic medium [7,35]. The antimicrobial activity of chitosan has been directly associated with its structural features, mainly the average degree of acetylation (DA) [36]. In this context, a decreased DA leads to higher density of positive charges of the chitosan chains and enhanced antimicrobial activity due to the electrostatic interactions involving the negatively charged moieties of biomolecules present in the cell wall and membranes. Nevertheless, sometimes further chemical functionalizations are required to achieve a more efficient self-assembled nanostructure, such as derivatization reactions that confer a permanent cationic character to chitosan. However, higher amounts of quaternized moieties can turn chitosan-based nanomaterials overly cytotoxic to healthy cells [37,38]. In this sense, the preparation of chitosan/PS formulations for aPDT seems to be an interesting strategy for synergistically achieving bacterial inactivation. For instance, nanocomposites of chitosan loaded with a PS agent, such as chloroaluminium phthalocyanine [39], methylene blue [40] or erythrosine [41], have improved antimicrobial action against planktonic and biofilm bacteria under controlled photoactivation. Recent work has demonstrated that low-molecular-weight chitosan has a higher aPDT effect against *H. pylori* when associated with methylene blue [42]. The effect of the chitosan’s molecular weight on its antimicrobial activity has been addressed with opposite tendencies against *E. coli* and *S. aureus*, suggesting that the chain length features distinct interaction mechanisms alongside the deacetylated moieties [43]. In addition, few approaches have considered the chelating ability of chitosan to prepare photocatalytic chitosan-metal oxide compounds aiming at antimicrobial applications [44,45]. ROS-mediated cell apoptosis has been promoted by chitosan/tripolyphosphate (TPP) nanoparticles internalized in tumor cells [46,47]. Even though the mechanism is still unclear, studies have demonstrated that chitosan NPs confers improved efficiency to trigger oxidative stress responses [46,47,48].

Based on the fact that chitosan preferably interacts with Gram-negative bacteria, while nonquaternized PPV is in turn more effective against Gram-positive bacteria, this study aimed to investigate aPDT potential of nanocomposites assembled with low-molecular-weight chitosan with a varied range of the DA, associated with cyano-substituted poly(*p*-phenylene vinylene) (CNPPV) against *E. coli* (Gram-negative) and *S. aureus* (Gram-positive) strains. Such a modified CNPPV structure is known to have higher electron affinity and increased ionization potential (low energy gap) when compared to PPV [49]. It is worth mentioning that, to the best of our knowledge, this type of photosensitive nanocomposite has never been prepared before, especially under the condition of DA variation, which was tested to verify how much the bacterial inactivation and photoinactivation would be affected by the amino moieties. Therefore, here we bring a systematic study that carries the proof-of-concept regarding the effect of the DA of chitosan, but also investigate the light-activated photocatalytic effect resulted from chitosan nanoparticles (NPs) and chitosan/CNPPV nanocomposites’ (NCPs) synergistic interaction when triggered by blue-light irradiation.

## 2. Results and Discussion

### 2.1. Spectroscopy Chacterization of Chitosans

The FT-Raman spectra of Ch samples are shown in Figure S1. Since the main variability was set as the average degree of acetylation, most differences were systematically related to vibrational modes from amino and acetamido groups. Indeed, the intensity of the bands corresponding to in-plane stretching vibration (υ) centered at 2932 cm^−1^ proportionally decreased with respect to that at 2889 cm^−1^ from Ch1 × 6 h to Ch3 × 6 h, confirming that the -CH_3_ group pertaining to acetamido moieties was accordingly partially and progressively removed as Ch1 × 6 h, Ch2 × 6 h and Ch3 × 6 h were considered. Analogously, the intensity of the band corresponding to υC=O decreased with respect to the in-plane bending vibration (δ) of NH_2_ at 1658 and 1598 cm^−1^, respectively. The spectral signal from 1500 to 1000 cm^−1^, remained mostly similar for all spectra, because it ranged distinct vibrational modes from the glucopyranose ring, as typically assigned in the fingerprint region of FTIR spectra of USAD chitosans [50], also confirming that the depolymerization process was ineffective for backbone disruption besides the β-linkages between vicinal pyranoses [51]. Among the vibrational bands ranged in this spectrum interval, that at 1460 cm^−1^ was referred to the υ, δ and out-of-plane bending (ω) of C-H and δOH, whereas 1413 cm^−1^ and 1375 cm^−1^ were related to symmetrical δ of -CH_3_, -CH_2_, -CH and -OH. The vibrational bands centered at 1323 cm^−1^ and 1263 cm^−1^ mainly corresponded to the hydrogen bonding δH…O, υC-C, υC-O and symmetrical stretching (ρ) of CH_2_, whereas those at 1147, 1117 and 1093 cm^−1^ were related to υC-O-C, υC-O, υC-CH_2_ and ρCH_3_. The 935 cm^−1^ and 897 cm^−1^ bands were referred to υC-N, which also revealed a similar tendency by varying the acetylation and stretching vibrations from glucopyranose ring, υ(ϕ), respectively [52].

**Figure 1:**
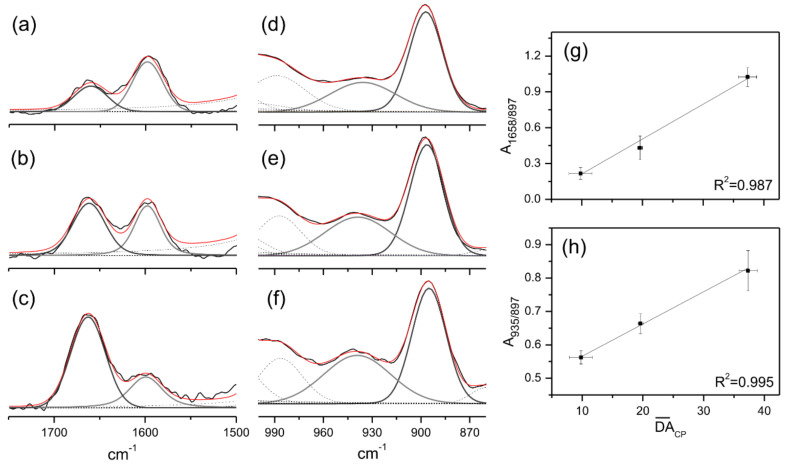
Deconvolution procedure of the assigned 1750–1500 cm^−1^ (**a**–**c**) and 1000–860 cm^−1^ (**d**–**f**) intervals of Ch3 × 6 h (**a**,**d**), Ch2 × 6 h (**b**,**e**) and Ch1 × 6 h (**c**,**f**) FT-Raman spectra using Voigt functions. Correlation between the normalized area and the calculated ${\bar{DA}}_{CP}$ values (**g**,**h**).

FT-Raman has often been rated as an accurate tool to quantitatively estimate structure modifications, not requiring a precise weighing of the sample. Here, we confirmed such a statement by a direct correlation between the calculated ${\bar{DA}}_{CP}$ and deconvoluted vibrational bands assigned to υC=O (1658 cm^−1^) and υC-N (935 cm^−1^) (Figure 1a–f). To ensure this procedure, we assumed the band centered at 897 cm^−1^ as an internal reference, once it was related to the unchanged structure of glucopyranose ring [52]. As a consequence, a linear relationship was built up using the integral ratios $A_{1658/897}$ and $A_{935/897}$ (Supplementary Materials) with ${\bar{DA}}_{CP}$ (Figure 1g,h), showing a high coefficient of determination (R^2^ > 0.98). Similarly to our previous findings [53], FT-Raman also figures as a fast and reliable spectroscopy technique, capable of predicting structural features of chitosan.

### 2.2. Physicochemical Characterization of Nanomaterials

**Figure 2:**
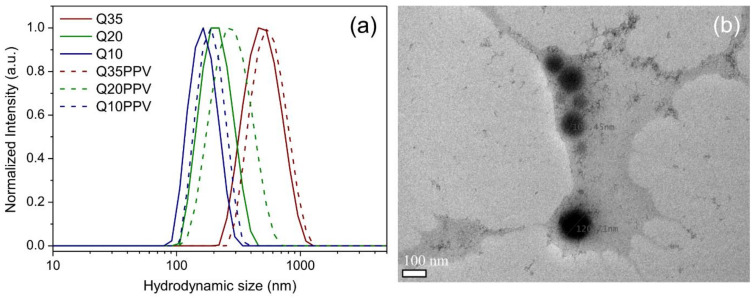
Hydrodynamic size distribution profile obtained by DLS of bare chitosan nanoparticles with varying DA (Q35, Q20 and Q10) and of the correspondent CN-PPV loaded particles (labelled as PPV) (**a**); representative TEM image from Q20 used as example at 100 nm of magnification, showing an average size and shape after drying (**b**).

The physical crosslinker sodium tripolyphosphate, TPP, was set to prepare functional materials in order to yield a nanoscale distribution profile and increased colloidal stability of the macromolecular suspension. As shown in Figure 2a, the DLS monomodal profile of chitosan-based NPs revealed the effect of chitosan’s DA on the resulting nanomaterials, which shifted accordingly to the content of acetamido moieties. The available sites for electrostatic interaction, i.e., amino groups of GlcN units of chitosan, are responsible for shrinking the structure after the interaction with an opposite charge and, once these amino groups increase from Q35 to Q10, the size distribution profile shifts to lower intervals, agreeing with the corresponding DAs. A proportional trend was observed also with the attachment of CNPPV, leading to a slightly higher size distribution with respect to the former and corresponding chitosan-based NPs, even considering that it represented about 1.5–2.0% (*w*/*w*) from the whole formulation. Once the electrostatic effect partially explained the size distribution profile, hence the maximum hydrodynamic size (*Dh_max_*), the stable nanostructure also remained with a positively charged surface, as described by the ZP values (Table S1), which also increased as much as the content of amino groups on the starting chitosan, before the attachment of CNPPV. In this sense, the chitosan’s DA mainly drove the nanosized range achieved due to the changes in the electrostatic interaction extension with TPP. Previous studies revealed that nonpermanent charged chitosans are able to partially sustain the cationic surface in pHs close to neutral, especially when participating on stable interactions with anionic species [37,38]. The present study demonstrates that such behavior can also be achieved with a higher DA and with the physical attachment of hydrophobic macrostructures with negative formal charges, such as CNPPV. Indeed, the latter seems to be responsible for effectively hiding the surface charge of the chitosan matrix, as the resulting nanocomposites showed a ZP closer to neutral (~2 mV), whereas chitosan nanoparticles were positively charged (10–28 mV) (Table S1).

Since the mechanism of the electrostatic interaction between chitosan and TPP is well known, involving the protonated amino groups and phosphates, respectively [54], it is expected that CNPPV would be trapped in the chitosan/TPP matrix as schematically represented in Figure S2 (Supplementary Materials), which may allow interchain interactions between CNPPV and chitosan chains, as similarly observed in our previous study [55].

Even though a considerable range of hydrodynamic sizes has been achieved, the drying process required for TEM measurements leads all nanomaterials to shrinking into smaller sizes [56], and accordingly, TEM of dried samples revealed diameters reaching around 100–200 nm, all in a rounded shape morphology (Figure 2b). These results also confirm that at higher DA values, as especially found in Q35 and Q35PPV samples, the swelling effect in aqueous medium was considerably higher with respect to that of the other nanomaterials, since their original hydrodynamic sizes reduced beyond half after drying. Such behavior indirectly reveals the hydrophobic feature carried by the acetamido moieties, which drives the nanomaterials’ formation to bigger sizes in an aqueous medium. Indeed, our previous study demonstrated a similar tendency resulted from a hydrophobically modified chitosan derivative [37]. It is important to note that the lower the DA, the higher the electrostatic interaction promoted by the amino moieties, which prevents larger chitosan/CNPPV associated structures from being formed. This decrease of the electrostatic interaction with the increase of the DA suggests that the acetamido moieties and their intrinsically higher hydrophobic nature act on repelling water molecules from the internal environment of nanomaterials, influencing directly the size of the nanoparticles.

**Figure 3:**
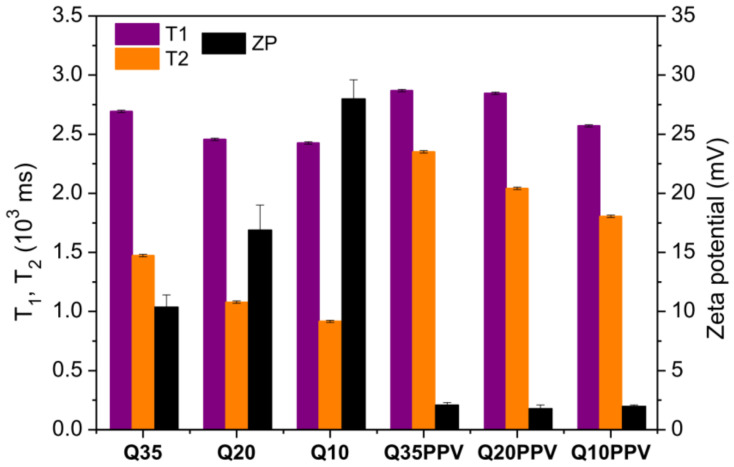
Longitudinal (T_1_) and transversal (T_2_) relaxation times calculated from TD-NMR signal data, and zeta potential values of NPs and NCPs.

The nanomaterials’ preparation was also evaluated by means of ^1^H NMR signal data. Whereas the ZP values explain the electrostatic behavior based on the resulting surface of nanomaterials, T_1_ and T_2_ values can provide a closer overview of their relationship within the first layers and the surrounding environment, once the majority of protons belong to the water molecules. In brief, the T_1_ and T_2_ relaxation times are dependent on the material viscosity or mobility of a given molecular component of the system. In colloidal dispersions or small molecules on low viscous fluids, T_1_ ≥ T_2_, due to the shorter time required for a given component to turnover its own symmetry axis (correlation time, *t_C_*). The gradual increase in viscosity leads to an increase in *t_C_* and a decrease in local field fluctuations at the Larmor frequency (*ω*_0_). The longitudinal relaxation efficiency, or relaxation rate 1/T_1_, reaches a maximum value in *t_C_*.*ω*_0_ ≈ 1 and, for higher intervals (*t_C_*.*ω*_0_ > 1), T_1_ > T_2_ for components with more restricted molecular motion, such as macromolecules and solid-state materials. Considering that the 1/T_2_ relaxation rate has no maximum value and decreases proportionally with molecular mobility, T_1_ >> T_2_ especially in solid-state materials [57,58]. Our data revealed that T_1_ was always higher than T_2_ (Figure 3) due to the intrinsically higher viscosity of the macromolecular systems, with such a difference being more evident for Q35, Q20 and Q10 samples than for the corresponding NCPs. Noticeably, T_2_ went the opposite way of ZP, which suggests that T_2_ was relatively sensitive to the electrostatic interaction among layers of water molecules and the remaining positively charged surface of nanomaterials, leading these surrounding water molecules to lose a certain degree of freedom or mobility around the NPs and NCPs. Indeed, in the case of chitosan/CNPPV, the lower surface charge density implies fewer sites available for interaction with water molecules, hence leading to higher values of T_2_ with respect to the ones without CNPPV. Therefore, TD-NMR indirectly confirmed the successful attachment between chitosan and CNPPV. All original TD-NMR curves can be found in Figure S3.

### 2.3. ROS Analysis

**Figure 4:**
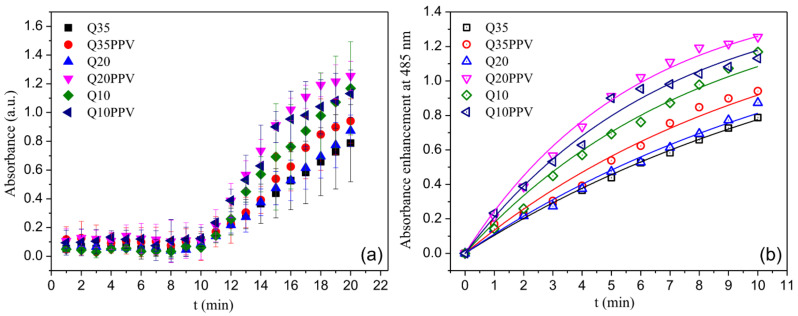
Absorbance profiles resulted from ROS production under blue-light irradiation during the whole analyzed interval as a function of time (**a**); 10 min range used for kinetic analyses (**b**). The solid lines presented in (**b**) were obtained fitting the data by using Equation (S5) shown in Section S.3 in the Supplementary Materials.

The ROS generation capability of NPs and NCPs under blue-light irradiation is shown in Figure 4a. The results demonstrate that the NPs did not generate ROS when kept under dark conditions. Differently, all NPs were able to produce ROS under blue-light irradiation, revealing distinct ROS production profiles depending on the NP formulation. Therefore, the kinetic analyses and resulting apparent rate constant (k_ROS_) were undertaken considering the last 10 min of the ROS measurements (i.e., it was considered with the data obtained when the NPs were under illumination), as can be seen in Figure 4b and Table 1, respectively.

Although absorption profiles did not show a great range of differentiation, the variability found was strongly dependent on the chitosan structure, featuring enhancements when the NPs were associated with CNPPV (Figure 4). These findings support that beyond the electronic cohesion established to form the NPs, both macromolecules acted as a singular nanomaterial when physically associated, in the way that ROS could be over-yielded by properly setting a given structure and composition. Here, the inherent light-activated capability of the conjugated polymer made CNPPV mainly responsible for electron transfers that in turn would trigger the major formation of oxide anions (O_2_^−^) and hydroxyl radicals (·OH). Alternatively, recent studies have also demonstrated that chitosan NPs can either produce or sustain ROS in certain microenvironments. Sarangapani et al. (2018) [48] have found that chitosan/TPP nanoparticles systems have greater potential to induce cell apoptosis which is associated with pro-oxidant activity, as compared to that of free chitosan. In healthy cells, these NPs induce low ROS and quench free radicals; however, an antioxidant imbalance takes place in tumor cells by depleting glutathione, an antioxidant regulator, hence leading to selective oxidative stress and ROS release. Further, Wang et al. (2018) [46] showed that chitosan NPs accelerates the accumulation of ROS, promoting a protective mechanism of autophagy on carcinoma cells. Similarly, Jiang et al. (2019) [47] confirmed that the internalization of chitosan NPs enables enhancing ROS-induced disorders on mitochondrial function. Noteworthy, none of these studies tested the ROS production potential of the NPs under light illumination and thus, the observed ROS were associated with a biological stimuli response. Even though the ROS generation mechanism promoted by chitosan NPs is still unclear, it seems reasonable that such a light-activated phenomenon is mainly driven or at least enhanced by non-acetylated moieties, once ROS production is clearly favored by a lowering DA, as we could demonstrate in this current study.

### 2.4. Bactericidal and Photobactericidal Activity

**Figure 5:**
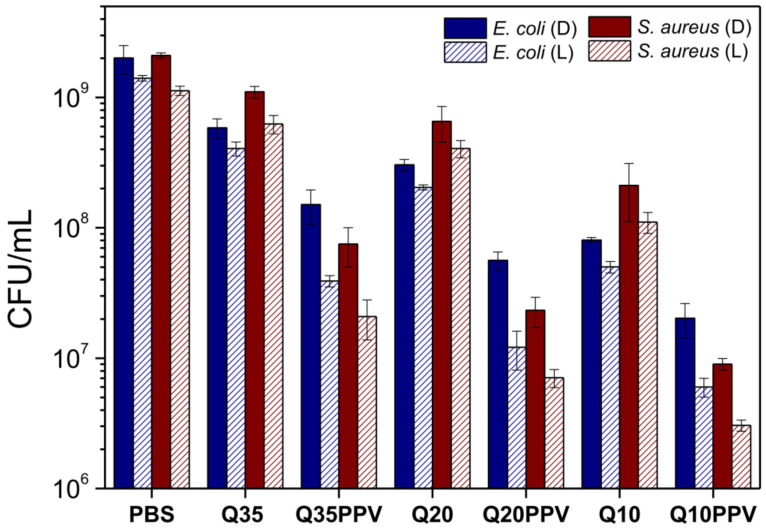
CFU mean values (±SD) of *E. coli* and *S. aureus* carried for non-irradiated (D) and irradiated (L) nanomaterials under 450 nm and 12 mW cm^−2^ for 1 h.

The bactericidal assay was performed under the same concentration for each nanomaterial (0.025 mg mL^−1^), previously determined by an MTT colorimetric assay. This experimental set up was chosen to properly count the colonies and explore the antimicrobial activity either related to the electrostatic and photobactericidal inactivation. MTT dye assay depends on the reduction of MTT salt by the bacteria’s mitochondrial dehydrogenase to form a purple-colored formazan product [59]. As shown in Figure 5, non-irradiated chitosan NPs demonstrated that their inactivation process was mainly addressed to the average quantity of non-acetylated moieties, systematically revealing increased antimicrobial inactivation by lowering the DA from Q35 to Q10. Such behavior has already been largely addressed elsewhere and it is generally accepted as a consequence of simultaneous mechanisms of action [60]. The first and most common one is due to the electrostatic interaction within ionic surfaces which facilitates the proximity between the NPs and bacteria interfaces, allowing the disruption of the cell wall/membrane and the leakage of cell cytoplasm. Previous work, related to the chitosan structure variability set in this study, reported enhanced cytotoxicity to an upgrade in the surface charge property of chitosan NPs [38]. Nevertheless, all chitosan NPs were quantitatively more efficient against *E. coli* than against *S. aureus*, probably due to the better binding affinity between chitosan NPs and *E. coli* in response to the electrostatic interaction. Despite the presence of negative anionic species on the surface of Gram-positive bacteria, it has been demonstrated that Gram-negative bacteria often exhibit more hydrophilicity, making them the most sensitive to chitosan [61]. In sequence, but at slower rates, the mechanism of penetration and binding of chitosan with nucleic acids after penetrating into the bacterial cells leads to the inhibition of DNA expression and protein synthesis. Although it is not fully elucidated, previous studies suggest that the visible damage caused on the cell membrane of *E. coli* and *S. aureus* is most likely due to the penetration of low-molecular-weight chitosans into the bacteria cytoplasm [62,63], also finding considerable enhanced potential for cell internalization as a chitosan/TPP nanoparticle-based system, compared to free chitosan [64]. Finally, the microbial rate growth could be lowered or even suppressed through the chelating ability of chitosan toward some metal ions and essential nutrients [36,65]. Beyond the bactericidal potential regarding the structure feature, several works have already reported increased performance from different kinds of polymeric NPs with dimensions below 200 nm [20,21,22,25,27]. At this point, we demonstrated a bactericidal inhibition responding to the structure’s electrostatic variability, which also influenced the size of the formed NPs and, consequently, the overall NPs’ bactericidal efficiency.

Chitosan NPs without CNPPV were also able to quantitatively increase its bactericidal inhibition after the exposure to irradiation with respect to that of the non-irradiated assay (Figure 5). Besides the well-known effect raised from the electrostatic interaction mediated by the DA, the irradiated experiments have successfully triggered these NPs for higher bactericidal activity. As far as we know, such behavior has not been addressed elsewhere under similar conditions, especially from chitosan-based nanomaterials without the association with any kind of photosensitizing agent. In this sense, these NP samples revealed photodynamic activity which might emerge from a similar process based on the triggered response of nanomaterials against carcinoma cells [46,47,48], with great remarks at lower DAs. According to these studies, chitosan can effectively sustain binding interactions at bacteria surfaces; however, the oxidative stress mechanism is most likely accelerated and accumulated after internalization in bacteria cytoplasmatic domains. In the case of *E. coli*, the enhanced bactericidal activity under dark conditions can be explained by the optimized disruption of the extra outer lipopolysaccharide membrane promoted by amino groups, allowing an efficient entrance of macromolecular content into the cytoplasmic environment. In contrast, the bactericidal activity improvement against *S. aureus* might be due to the successful binding with the open network of peptidoglycan and anionic teichoic and lipoteichoic acids, which enable a better interaction and cell wall disruption [33,66].

The formation of hydrosoluble and light-activated nanoscaled compounds associated with the polycationic feature of chitosan are definitely advantageous for inhibiting both Gram-positive and Gram-negative bacteria. ROS-mediated disruption may arise together with other feasible mechanisms, as mentioned before, fulfilling an important role in the inactivation process. The improved performance of chitosan/CNPPV bactericidal activity, concerning the ones without CNPPV, evidences a synergistic effect achieved through the effective attachment between macromolecules with a completely different nature. For all of them, the bactericidal activity is even more efficient after blue-light irradiation due to the generation of ROS by photodynamic action, this combined effect being greater as the content of amino groups increased, i.e., from Q35PPV to Q10PPV. This way, once the stronger binding favors the proximity of nanomaterials, the short-lived ^1^O_2_ species may be continuously formed closer to the bacteria neighborhood, leading to more efficient oxidative damage of cellular components [67]. Recent studies also propose that the macromolecular disaggregation inside the bacteria can slow nonradiative decay of the triplet state [32,68]. Since the lifetime of the triplet state is longer than that of an excited singlet, a slower process leads to an increasing ^1^O_2_ quantum yield, thus increasing the efficiency of ROS. These results confirms that a source of irradiation can evolve the efficiency of chitosan-based NPs against bacteria by improving ROS production, especially when associated with a conjugated polymer. Figure S4 presents a simplified schematic representation of the bactericidal and photobactericidal mechanism of action of NCPs against Gram-negative and Gram-positive bacteria.

## 3. Materials and Methods

### 3.1. Materials

The preparation process and structural characterization of low-molecular-weight chitosans (Ch) have already been detailed in our previous studies [51,53]. In brief, allomorph β-chitin, extracted from squid pens of *Doryteuthis* spp., was submitted to an ultrasound-assisted deacetylation process (USAD) under strong alkaline conditions, leaving a first chitosan sample named Ch1× as the main product. Two additional batches were conducted onto the last corresponding isolated product, resulting in chitosans Ch2× and Ch3× with a sequentially lower average degree of acetylation ($\bar{DA}$). Following that, these chitosans were dissolved in diluted aqueous acid and resubmitted to the ultrasound for 6 h at room temperature, promoting controlled depolymerization and resulting in the products described as Ch1 × 6 h, Ch2 × 6 h and Ch3 × 6 h. These chitosans presented an average weight molecular weight (${\bar{M}}_{w}$) in the range of 1.5–1.9 × 10^5^ g mol^−1^, obtained by size exclusion chromatography (SEC).

Sodium tripolyphosphate (TPP), dihydroethidium (DHE) and cyano-substituted poly(*p*-phenylenevinylene) (CNPPV) were purchased from Sigma-Aldrich (São Paulo, Brazil). The microbiological assays were performed using *E. coli* (ATCC 25922) and *S. aureus* (ATCC 25923) strains, 3-(4,5-dimethyltruiazol-2-yl)2,5-diphenyltetrazolium bromide) (MTT) (Sigma-Aldrich, São Paulo, Brazil), Plate Count Agar (PCA) medium (Acumedia, Neogen, Lansing, MI, USA) and Brain Heart Infusion Broth (BHIB) (KASVI, São Paulo, Brazil). All other chemicals used were of analytical grade.

### 3.2. Preparation of Chitosan-Based Nanomaterials

The nanomaterials (NPs and NCPs) were prepared based on the combination of two independent and well-established protocols [38,69,70] with some modifications. Initially, 16 mg of Ch samples was dispersed and kept under magnetic stirring in 8 mL of acetic acid 1% (*v*/*v*) for 24 h at room temperature. Sequentially, 1.0 mL of sodium tripolyphosphate (TPP) at 2.0 mg mL^−1^ in aqueous solution was slowly added, and the remaining solution was kept under constant stirring at 300 rpm at room temperature. The nanoparticles made from Ch1 × 6 h, Ch2 × 6 h and Ch3 × 6 h were named as Q35, Q20 and Q10, respectively. As previously reported and determined by high-resolution solid-state ^13^C CPMAS spectra (${\bar{DA}}_{CP}$) [51], average degrees of acetylation of 37.3 ± 1.5, 19.6 ± 0.4 and 9.8 ± 1.9% were obtained for Ch1 × 6 h, Ch2 × 6 h and Ch3 × 6 h, respectively. The preparation procedure of chitosan-based NCPs was repeated including the slow and simultaneous addition of 100 µL of CNPPV at 2.5 mg mL^−1^, diluted in tetrahydrofuran (THF). In each case, the open-batch solutions were left stirring overnight to allow the evaporation of the organic solvent. Finally, the nanosuspensions were refilled with distilled water until they reached 10 mL for concentration adjustment. The products were named Q35PPV, Q20PPV and Q10PPV, with respect to the ones prepared without CNPPV, respectively. The chitosan/CNPPV samples were filtered and centrifuged at 8000 rpm for 15 min, and all samples were stored around 4 °C. To ensure enough content of attached CNPPV to the NPs, 100 µL was dried in a desiccator and resuspended in THF. Diluted aliquots were analyzed using a UV-vis spectrometer, resulting 12.5 ± 0.4 µg mL^−1^, 16.3 ± 0.8 µg mL^−1^ and 18.1 ± 0.6 µg mL^−1^ of CNPPV for Q35PPV, Q20PPV and Q10PPV, respectively.

### 3.3. Characterization of Chitosans

#### Fourier Transform Raman Spectroscopy

FT-Raman spectra of solid chitosan samples were recorded in the 4000–100 cm^−1^ range with an operating resolution of 3.5 cm^−1^ using a Raman spectrometer model LabRAM HR Evolution from Horiba Scientific^®^ (Tokyo, Japan), equipped with a Czerny–Turner monochromator, a CCD detector and an Olympus confocal microscope (50 × objective) with a 532 nm laser. To avoid degradation effects, the laser’s power was set at 1 mW [71]. The spectrum regions between 1750 and 1500 cm^−1^ and 980 and 850 cm^−1^ were deconvoluted with Voigt functions using PeakFit 4.12 software to predict the average degree of acetylation from distinct spectrum intervals based on ${\bar{DA}}_{CP}$ as external reference [52].

### 3.4. Characterization of Nanomaterials

#### 3.4.1. Dynamic Light Scattering and Electrophoretic Behavior (DLS and ELS)

The average hydrodynamic size (*Dh*) and average zeta potential (ZP) of the NPs and NCPs were obtained by means of their dynamic light scattering and electrophoretic behavior, respectively. All measurements were performed through diluted aliquots (1:100) of NPs in an aqueous solution (10 mmol mL^−1^ of NaCl) using a Zetasizer Nano ZS (Malvern Instruments, Worcestershire, UK) equipped with a laser beam of He-Ne at 633 nm and an angle of 173°. The data acquisition was automatically repeated until reaching a stable monomodal distribution profile for accurate values of *Dh*, polydispersity index (PDI) and ZP [38].

#### 3.4.2. Transmission Electron Microscopy (TEM)

The average diameter and morphology of dried NPs and NCPs were evaluated by TEM images on a FEI Tecnai G2F20, Japan microscope with an acceleration voltage of 200 kV. The diluted NPs suspensions (10 mmol mL^−1^ of NaCl) were dripped and allowed to dry at room temperature on a film-coated copper microgrid (300 mesh) and then stained with a uranyl acetate 3% (*v*/*v*) solution [72].

#### 3.4.3. Time-Domain Nuclear Magnetic Resonance (TD-NMR)

^1^H TD-NMR measurements were carried out to confirm the preparation and composition of NPs and NCPs, taking into account the relationship between them and the surrounding water molecules [58]. The analyses were performed on a 11.3 MHz spectrometer (0.27 T for ^1^H resonance frequency) SLK-200 (SpinLock, Argentina) using a 30 mm probe at 30 °C. The determination of longitudinal (T_1_) and transversal (T_2_) relaxation times performed using the Continuous wave-free precession (CWFP-T_1_) [73] and the Carr–Purcell–Meiboom–Gill (CPMG) [74] pulse sequences, respectively. The CPMG sequence was executed using 90° and 180° pulses of 9.0 and 18.5 µs, respectively, and echo times of 1.0 µs, with a total of 20,000 echoes, a recycle delay of 5 s and 256 scans. The CWFP-T_1_ sequence was performed using a 180° pulse of 18.5 ms and a 10° pulse of 1.0 µs, with an interpulse delay of 0.3 µs and 60,000 acquisition points, a recycle delay of 1 s and 128 scans. The CWFP-T_1_ data was treated with a previously described methodology [75] to reduce the data size and increase the signal-to-noise ratio, resulting in the T_1_ curve with one hundred data points.

### 3.5. Bactericidal and Photobactericidal Assays

The colorimetric assay commonly used for measuring cell metabolic activity based on the cellular reduction of MTT salts was preliminary carried out for screening the activity of each NPs and NCPs in the concentration interval 0.01–1.0 mg mL^−1^, aiming to determine their concentration to be used in a colony growth assay, based on the spread plate method (PCA) adapted from previous studies [8,76]. Thus, the nanomaterials were split into two independent groups: the first one was kept in the dark (non-irradiated group) and the other was submitted to blue-light irradiation (irradiated group). The irradiated group was incubated under dark conditions for 30 min prior to irradiation. The bacteria were initially dispersed in BHIB media and incubated for 24 h at 37 °C. A final bacterial concentration of about 1.5 × 10^8^ CFU mL^−1^ was obtained and further diluted with NPs, NCPs and PBS and used as negative control in 96-well polystyrene plates. The MTT incubation was set for 4 h at 37 °C. This experiment was repeated, submitting an equivalent 96-well plate to irradiation (450 nm) at 12 mW cm^−2^ for 1 h before incubating. After the incubation, all testing content was diluted with isopropanol, raising contrast within those wells with dissolved formazan. Afterwards, a given concentration of each nanomaterial was sampled with an equivalent bacteria inoculum concentration and also submitted to the same irradiation conditions. The NPs and NCPs suspensions with and without previous irradiation were diluted in PBS plated on sabourad dextrose agar (SDA). The total number of bacteria, i.e., colony-forming units (CFU), was counted after 18 h of incubation at 37 °C. All bioassays were performed in triplicate in three independent experiments.

### 3.6. Reactive Oxygen Species Experiments (ROS)

The determination of ROS produced by the nanomaterials was investigated using a non-fluorescent marker, DHE, and performed under blue-light irradiation. Since the oxidation reaction among the marker and ROS provides ethidium molecules as a highly fluorescent product, its presence allows the detection and quantification of oxidative species [76,77]. All measurements were carried out in a 1 cm pathlength quartz cuvette containing 2 mL of the nanomaterials in an aqueous solution with DHE at 0.34 mM. The production of ROS was monitored in dark conditions in the first 10 min, then, real-time exposure surrounded by a blue-light source of irradiation (450 nm) at 12 mW cm^−2^ was turned on and applied for 10 more minutes to promote the photochemical reaction. The kinetics of ROS production were monitored using a Lambda 265 model spectrometer (PerkinElmer), and the rate constant $k_{ROS}$ was determined as described in a previous study [77]. However, due to the fluorescence feature of CNPPV, an absorbance instead of fluorescence signal was used to calculate the values of $k_{ROS}$. Previous findings confirm that the absorption experiments of ethidium salt, which is assigned to the absorption peak corresponding to π → π* transitions from N(C_2_H_5_) groups, can be advantageous over the fluorescence ones, since the latter could bring unavoidable overlapped profiles with other fluorescent species, such as the case of CNPPV [78].

## 4. Conclusions

Herein, we designed and produced novel nanocomposites (NCPs) based on chitosan/CNPPV, employing chitosans displaying different variable average degrees of acetylation. The nanomaterials’ production was carried out on the basis of the urgent demands to develop innovative approaches to fight against resistant bacteria. Considering the bactericidal potential of both chitosan and conjugated polymers, we combined them to obtain nanobased materials with synergistic antimicrobial activity against Gram-positive and Gram-negative bacteria, with an additional potential of being light-activated (i.e., photoantimicrobial activity). Stable nanomaterials were found as confirmed by the surface charge tendency ascribed by the zeta potential values and relaxation time T_2_. Meaningful variations on both parameters served as a reliable confirmation of successful chitosan/CNPPV attachment. Hence, the electrostatic behavior of chitosan that resulted from the deacetylated moieties was assigned as an important driven force that provided such stability. In general, the overall features and antimicrobial performance of the prepared NPs and NCPs were straightly dependent on the content of chitosan’s non-acetylated groups. Indeed, ROS generation was triggered by blue-light irradiation, with enhanced the performance at lower DA values. The ROS generation capability of chitosan-based NPs was demonstrated when prepared with or without CNPPV, which was shown to be proportionally driven by the content of non-acetylated groups. In summary, the reduction of the degree of acetylation improved the antimicrobial activity against Gram-positive and Gram-negative bacteria, in which the highest bacterial activity was achieved by ROS-mediated chitosan/CNPPV nanocomposites under blue-light irradiation.

## Figures and Tables

**Table 1:** Rate constant of ROS production under blue-light irradiation. $k_{a}$ and $k_{ROS}$ values were obtained as presented in Section S.3 in the Supplementary Materials.

Samples	$k_{a}(10^{-3}\;s^{-1})$	$k_{ROS}(M^{-1}s^{-1})$
Q35	1.19 ± 0.02	3.49 ± 0.06
Q35PPV	1.54 ± 0.05	4.52 ± 0.14
Q20	1.27 ± 0.25	3.73 ± 0.73
Q20PPV	2.91 ± 0.07	8.56 ± 0.21
Q10	2.07 ± 0.06	6.07 ± 0.18
Q10PPV	2.47 ± 0.08	7.26 ± 0.22

## Data Availability

Not applicable.

## Supplementary Materials

The following supporting information can be downloaded at: https://www.mdpi.com/article/10.3390/ijms232012519/s1.
