# Supplementary material for: Antimicrobial and Photoantimicrobial Activities of Chitosan/CNPPV Nanocomposites
Source: Int J Mol Sci. 2022 Oct 19;23(20):12519. doi: 10.3390/ijms232012519 (PMC9604068; doi:10.3390/ijms232012519)
Supplement: Supplementary file 1 [file ijms-23-12519-s001.zip › ijms-1960177-supplementary.pdf]

Supplementary Material

Article

Antimicrobial and photoantimicrobial activities of chitosan/CNPPV nanocomposites

William M. Facchinatto [1,*], Leandro O. Araujo [1], Tiago B. Moraes [2], Thais F. Abelha [1], Thalita H. N. Lima [1], Danilo M. dos Santos [3], Sérgio P. Campana-Filho [4], Luiz A. Colnago [3], Anderson R. L. Caires [1,*]

[1] Laboratory of Optics and Photonics, Institute of Physics, Federal University of Mato Grosso do Sul, Ave. Costa e Silva s/n, Zip Code 79070-900, Campo Grande, MS, Brazil;
[2] Department of Biosystems Engineering, "Luiz de Queiroz" College of Agriculture, University of São Paulo, Ave. Pádua Dias 11, Zip Code 13418-900, Piracicaba, SP, Brazil;
[3] Brazilian Corporation for Agricultural Research, Embrapa Instrumentation, St. XV de Novembro 1452, Zip Code 13560-970, São Carlos, SP, Brazil;
[4] São Carlos Institute of Chemistry, University of São Paulo, Ave. Trabalhador são-carlense 400, Zip Code 13560-590, São Carlos, SP, Brazil;
* Correspondence: williamfacchinatto@alumni.usp.br (W.M.F.); anderson.caires@ufms.br (A.R.L.C.).

S.1. Spectroscopy characterization of chitosans

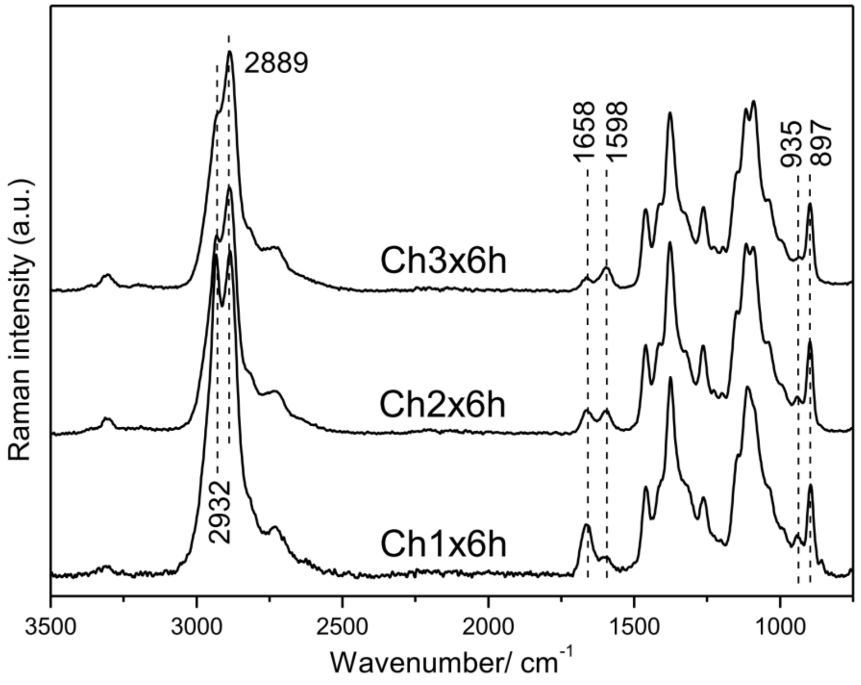

Figure S1. FT-Raman spectra of depolymerized Ch samples.

Linear dependence of Raman bands with the predicted average degree of acetylation calculated from ^{13}C CPMAS spectra ($\overline{DA}_{CP}$):

$$\boldsymbol{A_{1658/897} = -0.079 + 0.029 \times \overline{DA}_{CP}} \quad \textbf{Eq. (S1)}$$

$$\boldsymbol{A_{935/897} = 0.469 + 0.010 \times \overline{DA}_{CP}} \quad \textbf{Eq. (S2)}$$

S.2. Physicochemical characterization of NPs

Table S1. Average hydrodynamic size of maximum intensity, Dh_{max} (nm), polydispersity index (PDI), zeta potential, ZP (mV), and longitudinal (T_1) and transversal (T_2) relaxation times of NPs

NPs	Dh_{max} (nm)	PDI	ZP (mV)	T_1 (ms)	T_2 (ms)
Q35	464.9 ± 18.1	0.099 ± 0.021	10.4 ± 1.0	2693 ± 10	1474 ± 10
Q35PPV	567.2 ± 26.7	0.198 ± 0.044	2.1 ± 0.2	2870 ± 10	2352 ± 10
Q20	200.9 ± 12.3	0.061 ± 0.019	16.9 ± 2.1	2456 ± 10	1079 ± 10
Q20PPV	256.0 ± 20.1	0.118 ± 0.036	1.8 ± 0.3	2847 ± 10	2042 ± 10
Q10	158.6 ± 9.2	0.057 ± 0.011	28.0 ± 1.6	2426 ± 10	917 ± 10
Q10PPV	183.7 ± 12.9	0.033 ± 0.010	2.0 ± 0.1	2571 ± 10	1806 ± 10

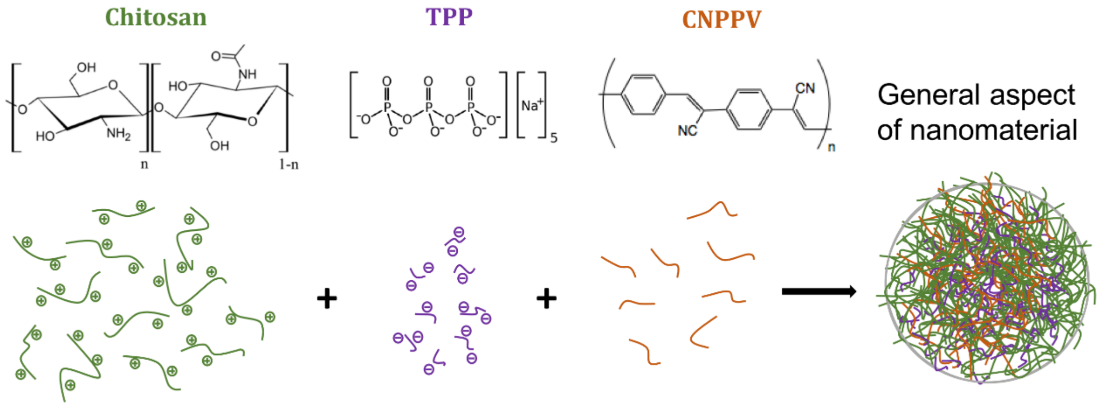

Figure S2. Schematic representation of the nanomaterial formation driven by the electrostatic interaction between chitosan and TPP, and CNPPV as a third species trapped in the chitosan-TPP matrix.

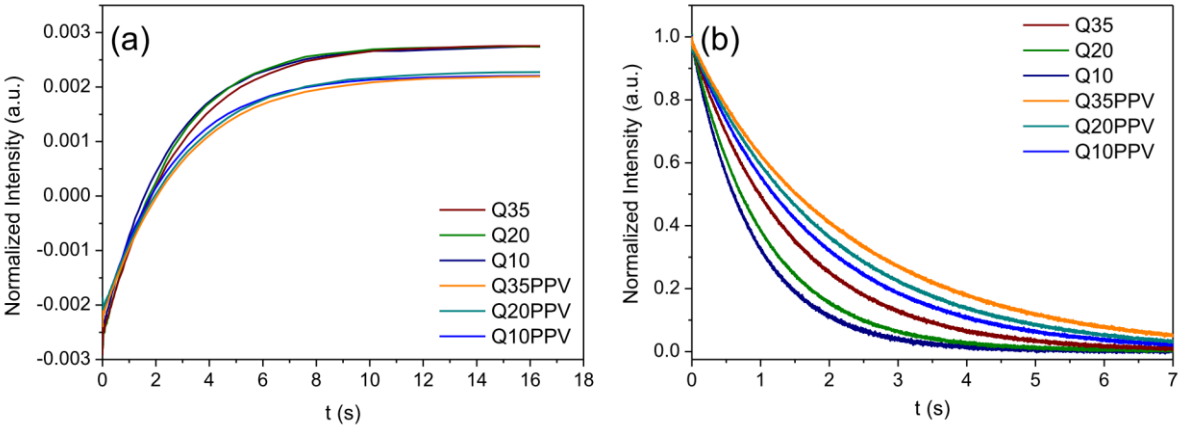

Figure S3. CWFP-T_1 (a) and CPMG (b) curves acquired by a TD-NMR benchtop equipment from NPs suspensions at the final concentration.

S.3. Kinetic analysis of the ROS production (reaction between DHE and ROS)

The rate constant of ROS production was estimated by kinetic analysis of the absorbance products generated by the interaction between DHE and ROS. DHE was used at a saturating concentration for the absorbance measurements, and it was assumed the formation rate of new absorbance products [A] equal to the one for ROS generation by nanoparticles under blue-light irradiation, as follows:[1]

$$\boldsymbol{DHE + ROS \overset{k}{\rightarrow} A}$$

Consequently, the rate of ROS production can be written as:

$$-\frac{d[ROS]}{dt} = k_{ROS}\,[DHE][ROS] \qquad \textbf{Eq. (S3)}$$

where k_{ROS} is the apparent rate constant of ROS production (*i.e.*, the apparent rate constant for the reaction of DHE with ROS), with $[ROS] \propto A$ and $[DHE] = 0.34\ mM$. Therefore, Eq. S4 can be rewritten as:

$$-\frac{dA}{dt} = k_a\,A \qquad \textbf{Eq. (S4)}$$

$$A = \sigma(1 - e^{-k_a t}) \qquad \textbf{Eq. (S5)}$$

where $k_a = k_{ROS}[DHE]$

S.4. Schematically representation of the bactericidal and photobactericidal activities of nanomaterials against Gram-negative and Gram-positive bacteria.

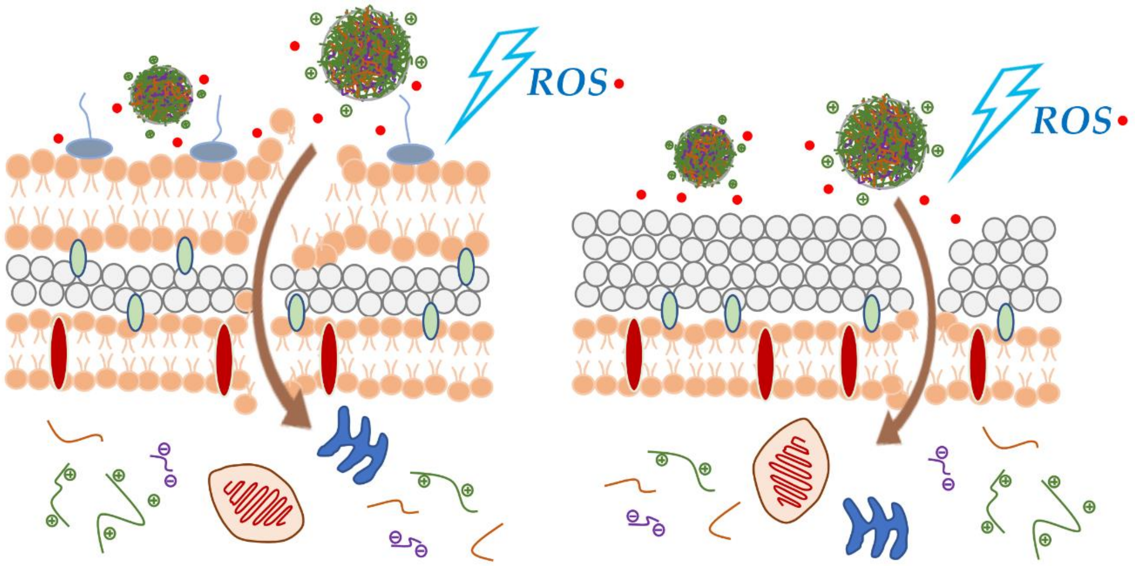

Figure S4. Proposed models of action of chitosan/CNPPV nanocomposites against Gram-negative outer membrane (left) and Gram-positive peptidoglycan layer (right) bacteria. The presence of surface charges and light-activated ROS are responsible for the interaction, disruption and oxidative degradation of outer layers, including the cytoplasmatic membranes. The scheme also shows the NCP's disaggregation and the presence of organelles, which are exposed to a similar mechanism of action.
